# RelB/NF-*κ*B links cell cycle transition and apoptosis to endometrioid adenocarcinoma tumorigenesis

**DOI:** 10.1038/cddis.2016.309

**Published:** 2016-10-06

**Authors:** Qiu-Lin Ge, San-Hong Liu, Zhi-Hong Ai, Min-Fang Tao, Li Ma, Shan-Yun Wen, Miao Dai, Fei Liu, Han-Shao Liu, Rong-Zhen Jiang, Zhuo-Wei Xue, Yu-Hang Jiang, Xiao-Hua Sun, Yi-Ming Hu, Yong-Xu Zhao, Xi Chen, Yu Tao, Xiao-Lu Zhu, Wen-Jing Ding, Bing-Qing Yang, Dan-Dan Liu, Xiao-Ren Zhang, Yin-Cheng Teng

**Affiliations:** 1 Department of Obstetrics and Gynecology, The Sixth People's Hospital affiliated with Shanghai Jiao Tong University, Shanghai, China; 2 Key Laboratory of Stem Cell Biology, Institute of Health Sciences, Shanghai Institutes for Biological Sciences, Chinese Academy of Sciences and Shanghai Jiao Tong University School of Medicine, Shanghai, China; 3 Department of Obstetrics and Gynecology, Shanghai Songjiang District Central Hospital, Shanghai, China; 4 Department of Gynecology, Obstetrics and Gynecology Hospital of Fudan University, Shanghai, China

## Abstract

Dysfunction of nuclear factor-*κ*B (NF-*κ*B) signaling has been causally associated with numerous human malignancies. Although the NF-*κ*B family of genes has been implicated in endometrial carcinogenesis, information regarding the involvement of central regulators of NF-*κ*B signaling in human endometrial cancer (EC) is limited. Here, we investigated the specific roles of canonical and noncanonical NF-*κ*B signaling in endometrial tumorigenesis. We found that NF-*κ*B RelB protein, but not RelA, displayed high expression in EC samples and cell lines, with predominant elevation in endometrioid adenocarcinoma (EEC). Moreover, tumor cell-intrinsic RelB was responsible for the abundant levels of c-Myc, cyclin D1, Bcl-2 and Bcl-xL, which are key regulators of cell cycle transition, apoptosis and proliferation in EEC. In contrast, p27 expression was enhanced by RelB depletion. Thus, increased RelB in human EC is associated with enhanced EEC cell growth, leading to endometrial cell tumorigenicity. Our results reveal that regulatory RelB in noncanonical NF-*κ*B signaling may serve as a therapeutic target to block EC initiation.

Endometrial carcinoma (EC) ranks as one of the most frequent gynecological malignancies, and the incidence continues to rise.^1^ EC is broadly categorized into two subtypes, type I endometrioid adenocarcinoma (EEC) and type II non-EEC (NEEC), based on histological features, hormone receptor status and grade.^2^ EEC, the most common subtype, is a low-grade, hormone receptor-positive tumor that typically coexists with or is preceded by endometrial hyperplasia. The clinical outcome of EEC is promising if identified at an early stage. NEEC, which accounts for 10–20% of EC cases, is defined as a high-grade, hormone receptor-negative, *TP53*-mutated neoplasm that exhibits an increased probability of metastasis and a relatively unfavorable prognosis. Advances in EC diagnosis currently allow for early intervention with surgery or adjuvant radiochemotherapy tailored to histology, which has markedly improved the overall survival of EC patients.^3^ However, EEC patients with advanced stages present with more aggressive tumors and tend to have a poorer prognosis than NEEC patients.^4^ Similar trends are noted for patients diagnosed under 40 years of age who seek to preserve fertility; remarkably, this population is quickly growing.^5^ Confronted with this situation, non-invasive targeted therapeutic strategies with minimal side effects are urgently needed.^6^

Inflammation is noted in almost all malignancies, even during the initial stages of cancer development.^7^ Chronic inflammation is an important oncogenic mechanism driving the occurrence of EC, and the role of inflammation in EEC has been well characterized.^8^ Nuclear factor-*κ*B (NF-*κ*B) is a well-known transcription factor involved in a wide variety of cellular processes, such as inflammation, apoptosis and proliferation.^5, 9^ NF-κB activation is triggered via two major pathways: the RelA-mediated classic pathway and the RelB-mediated alternative pathway.^10^ NF-κB family members, such as p65/RelA and p52/RelB, have been detected in EC, and their expression is augmented by hypoxia.^11^ Based on the above findings, it is likely that NF-κB signaling is involved in malignant uterine lesions. However, the exact functions of NF-κB signaling components in EC remain largely unknown, although the development of specific therapeutics targeting vital NF-κB subunits may afford cancer patients considerable benefit.

In this study, we detected abnormally high expression of alternative RelB/NF-κB in clinical EC samples. In particular, RelB protein was significantly upregulated in both the International Federation of Gynecology and Obstetrics (FIGO) stage I and stage II or III EEC cases. Moreover, inhibition of RelB inhibited endometrioid cancer cell growth both *in vitro* and *in vivo*. Comparison studies revealed a critical role for RelB in maintaining cell cycle progression, and RelB was found to protect against apoptosis in certain EEC cell lines. Mechanistic studies further showed that increased RelB induced the expression of genes positively associated with G1/S transition, such as c-Myc and cyclin D1, whereas factors inversely related to apoptosis, such as Bcl-2 and Bcl-xL, exhibited reduced expression in response to decreases in RelB. Conversely, p27 and p21 expression was amplified by RelB inhibition. Together, our results suggest that the alternative NF-κB pathway is a crucial factor in EEC tumorigenesis and that RelB represents a considerable molecular target for EEC.

## Results

### Alternative RelB/NF-*κ*B is elevated in EEC regardless of stage

**Figure 1:**
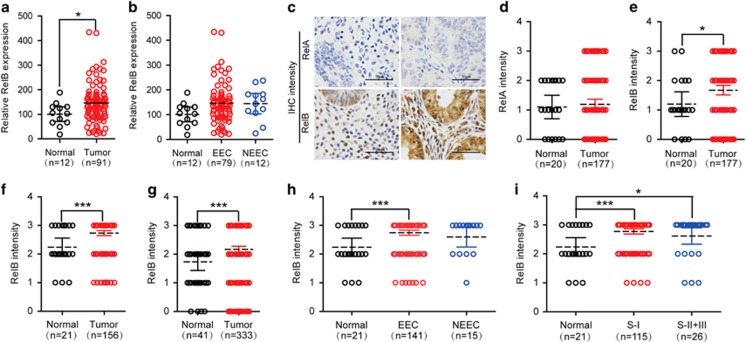
A significant increase in the alternative RelB/NF-*κ*B is noted in EEC. (**a**) GEO gene array data for RelB expression in EC (*n*=91) patients *versus* normal controls (*n*=12). (**b**) GEO gene array analysis for RelB expression in EEC (*n*=79) and NEEC (*n*=12) compared with controls (*n*=12). (**c-e**) Photographs (**c**) and quantification of RelA (**d**) and RelB (**e**) IHC staining of EC (*n*=177) and controls (*n*=20) from TMA-1, demonstrating significant upregulation of RelB protein in EC specimens. The IHC score was evaluated blindly by combining the percentage of staining intensity with positive staining as follows: 0 (negative, no positive cells), 1 (weak, 0–10%), 2 (moderate, 10–60%) and 3 (strong, >60%), the same below. (**f**) Quantification of RelB IHC staining in EC (*n*=156) *versus* normal controls (*n*=21) from TMA-2. (**g**) Combined analysis of RelB IHC staining in EC (*n*=333) compared with controls (*n*=41) from TMA-1 and TMA-2. (**h**) Stratified quantification of RelB expression in EEC (*n*=141) and NEEC (*n*=16) compared with normal controls (*n*=21) in TMA-2, displaying elevated RelB in EEC but not NEEC. (**i**) Hierarchical quantification of RelB IHC staining in relation to the FIGO stage of EEC from TMA-2, revealing that increased RelB is not clearly correlated with the FIGO stage of EEC. All analyzed data are presented as a scatter dot blot, with a line showing the mean and 95% CI, *P<0.05, ***P<0.001

To explore whether the NF-*κ*B pathway influences human EC, we first assessed GEO data sets. We identified an upregulation of the alternative RelB/NF-*κ*B in EC compared with normal controls, and this increased expression manifested a clear trend toward EEC (Figures 1a and b). To confirm this observation, we performed IHC-based TMA analysis. We first analyzed the expression of the canonical RelA/NF-*κ*B and alternative RelB/NF-*κ*B in tissue sections of 177 EC samples in TMA-1. The results showed significantly enhanced expression of RelB, but not RelA, in EC tumor cell specimens (Figures 1c and e). Moreover, a similar elevation in RelB was observed in another independent sample of 156 EC tissue sections in TMA-2, regardless of whether these data were combined with those of TMA-1 (Figures 1f and g). To examine the association between upregulated RelB and tumor stage and histological type, we further analyzed the TMA-2 data and correlated RelB expression with clinicopathological parameters. These results revealed that increased RelB expression was not significantly associated with the FIGO EEC stage (Figures 1h and i). In addition, clinical relevance of RelB expression with other factors, such as age, NEEC, lymphatic metastasis or vascular invasion (Table 1), was not observed. These results indicate that noncanonical RelB/NF-*κ*B is activated in the early development of EEC.

### Enhanced RelB/NF-*κ*B is essential for the growth of EEC cells

**Figure 2:**
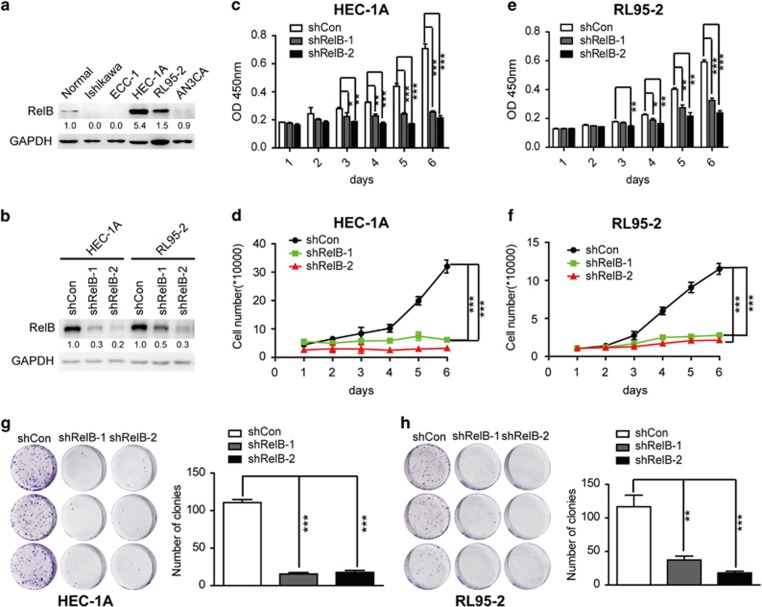
RelB depletion inhibits HEC-1A and RL95-2 cell growth. (**a**) RelB protein levels in different human EEC cells. (**b**) The protein level of RelB in HEC-1A and RL95-2 cells expressing shCon, shRelB-1 or shRelB-2. (**c** and **e**) Cell counting assay using the CCK-8 kit of HEC-1A (**c**) and RL95-2 (**e**) cells expressing shCon and shRelB-1 and -2. RelB knockdown significantly reduced cell proliferation. In all, 1 × 10^3^ cells per well were seeded in a 96-well plate, and cell viability was measured as the optical density at 450 nm (OD450) each day for 6 days. (**d** and **f**) The growth curve of HEC-1A (**d**) and RL95-2 (**f**) cells with shCon, shRelB-1, or shRelB-2. In total, 1 × 10^4^ cells per well were seeded in a 12-well plate, and cell numbers were assessed each day for 6 days. (**g** and **h**) The colony formation assay of HEC-1A (**g**) and RL95-2 (**h**) cells expressing shCon, shRelB-1 or shRelB-2. Colonies were counted after 10 days. RelB inhibition reduced the colony formation ability. All data are presented as the mean±S.D. of three independent experiments, *P<0.05, **P<0.01, ***P<0.001

Endometrial hyperplasia is an early event in EEC tumorigenesis and is the primary cause of onset of this tumor.^2^ Therefore, we hypothesized that the alternative RelB/NF-*κ*B may operate as an oncogenic insult in the area of endometrial hyperactive proliferation. To assess this possibility, we first examined RelB expression in five EEC cell lines using primary normal endometrial cells as a control. RelB was increased in HEC-1A and RL95-2 cells compared with the other cells (Figure 2a). RelB expression was then knocked down for proliferation studies through the use of short-hairpin RNA (shRNA) (Figure 2b). The proliferation rates of shRelB-expressing HEC-1A and RL95-2 cells were then compared with control cells (expressing an empty vehicle). As shown in Figures 2c and h, both EEC cell lines expressing downregulated RelB displayed a reduced proliferation rate compared with cells expressing the empty vehicle. In order to exclude the effects of other NF-*κ*B signaling pathways, we detected the protein expression levels of RelA, NF-*κ*B1, NF-*κ*B2 or c-Rel in the RelB knockdown HEC-1A and RL95-2 cells. The results showed that the protein expression levels of RelA, NF*κ*B1, NF*κ*B2 or c-Rel in both cytoplasm and nuclear were not significantly changed in the RelB knockdown HEC-1A and RL95-2 cells (Supplementary Figure S1). These results indicated that the alternative RelB/NF-*κ*B is indeed functional in the context of hyperplasia and tumor initiation in EEC.

### The *in vivo* growth of RelB-deficient EEC cells is substantially reduced

**Figure 3:**
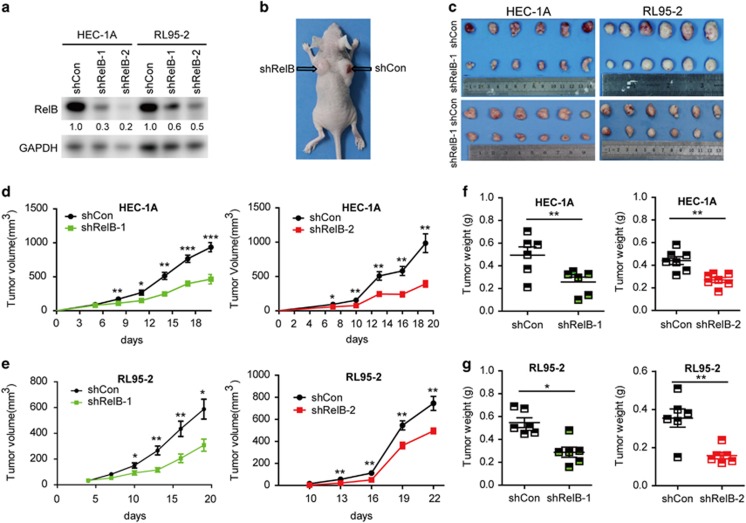
RelB reduction suppresses EEC cell growth *in vivo*. (**a**) RelB protein levels in HEC-1A and RL95-2 cells with shCon, shRelB-1 or shRelB-2. (**b**) Image of subcutaneous injection with HEC-1A and RL95-2 cells expressing shCon, shRelB-1 or shRelB-2. (**c**) Tumor growth in mice injected with HEC-1A (left) and RL95-2 cells stably expressing shCon, shRelB-1 or shRelB-2 (*n*=6 for each group). (**d** and **e**) Tumor volume in mice injected with HEC-1A (**d**) and RL95-2 (**e**) cells stably expressing shCon, shRelB-1 or shRelB-2 (*n*=6 for each group). (**f** and **g**) Tumor weight in mice injected with HEC-1A (**f**) and RL95-2 (**g**) cells stably expressing shCon, shRelB-1 or shRelB-2 (*n*=6 for each group). All data are presented as the mean±S.E.M., *P<0.05, **P<0.01, ***P<0.001

To validate the functionality of RelB, we next used an alternative experimental strategy. We established a subcutaneous xenograft model in nude mice using EEC cells with knocked down RelB expression or the empty vector control (Figures 3a and b). Consistent with our previous results, RelB-deficient EEC cells exhibited diminished proliferation in the subcutaneous xenograft model (Figures 3c and g). This finding confirms that RelB is functionally active and can bestow a growth advantage to EEC cells.

### Tumor cell-associated RelB facilitates tumor growth through enhanced G1-to-S entry and protection against apoptosis

**Figure 4:**
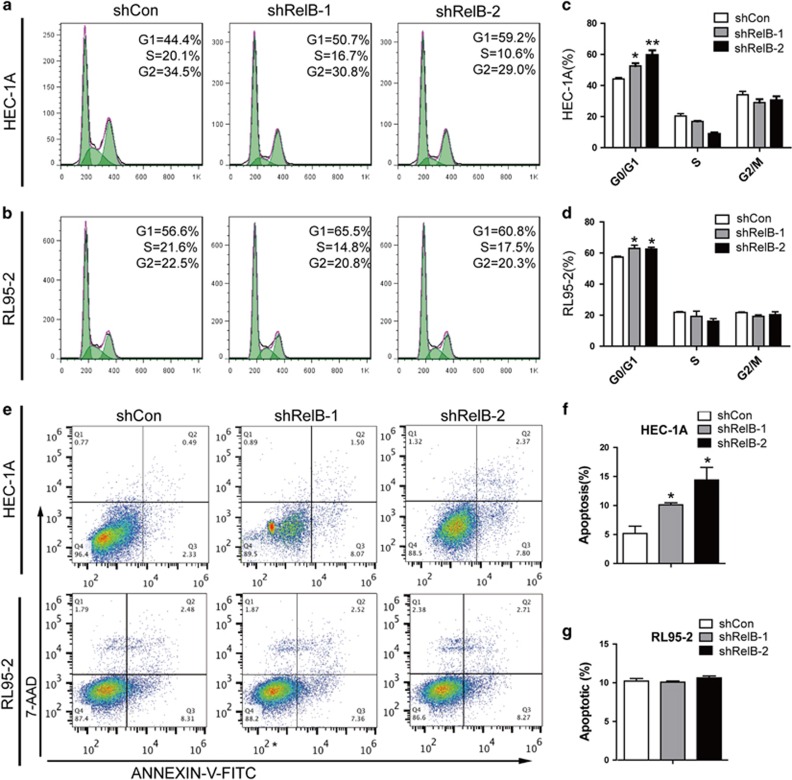
RelB ablation increases the G1 cell population and promotes apoptosis. (**a** and **b**) Profiling of the cell cycle distribution of HEC-1A (**a**) and RL95-2 (**b**) cells transiently expressing shCon, shRelB-1 or shRelB-2 based on DNA staining with propidium iodide. (**c** and **d**) Quantitative analyses of the cell cycle distribution as described in **a** and **b**, assessed by two-sided Student's *t*-test. (**e-g**) Flow cytometry profiling (upper panel: HEC-1A; lower panel: RL95-2) and quantification (**f** for HEC-1A and **g** for RL95-2) of Annexin-V/7-AAD-stained cells transiently expressing shCon, shRelB-1 or shRelB-2. All data represent the mean±S.D. of three independent experiments, *P<0.05, **P<0.01

The cell cycle and apoptosis are quintessential regulatory components of NF-κB signaling in human cancer.^12^ Thus, we investigated whether alterations in these processes could explain the strikingly reduced tumor cell growth observed in RelB-deficient EEC cells. Cell cycle distribution analysis revealed that both HEC-1A and RL95-2 cells with downregulated RelB exhibited a significant increase in populations in the G0/G1 phase compared with empty control cells (Figures 4a and d). Regarding apoptosis, increased levels of apoptosis were demonstrated in RelB-abated HEC-1A cells compared with controls, whereas no discrepancies in apoptosis were observed upon the downregulation of RelB in RL95-2 cells (Figures 4e and f). Thus, advanced G1-to-S progression and the prevention of apoptosis act in concert to fuel accelerated tumor growth in the presence of tumor cell-intrinsic RelB.

### RelB depletion results in G0/G1 arrest and apoptosis via essential genes implicated in NF-*κ*B, cell cycle and apoptosis pathways

**Figure 5:**
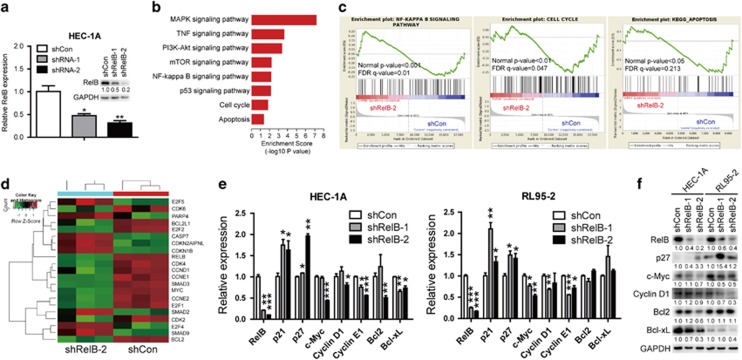
Molecular phenotype of HEC-1A cells with reduced RelB expression. (**a**) RelB mRNA and protein levels in HEC-1A cells with shCon, shRelB-1 or shRelB-2. (**b**) The top 10 most significant pathways that were enriched with shRelB. (**c**) Gene set enrichment analyses for signaling cascades of NF-*κ*B-enriched (left), cell cycle-enriched (middle) and apoptosis-enriched gene expression in HEC-1A cells. (**d**) Hierarchical clustering of essential genes implicated in NF-*κ*B, cell cycle and apoptosis pathways. Heatmap demonstrates the correlation of each gene with RelB downregulation targeting these three pathways in HEC-1A cells. Red, amplification; green, inhibition. (**e** and **f**) Verification of RelB target genes at the mRNA (**e**) and protein (**f**) levels by quantitative real-time PCR and WB assays, respectively. Upregulation of p21 and p27 was verified; downregulation of c-Myc, cyclin D1, cyclin E1, Bcl-2 and Bcl-xL was confirmed. All data represent the mean±S.D., *P<0.05, **P<0.01, ***P<0.001

The above results showed that tumor cells undergo G0/G1 arrest and apoptosis upon silencing of endogenous RelB. Thus, to further characterize the mechanisms involved in carcinogenesis stimulated by RelB activation, we conducted gene expression arrays to identify gene sets that were enriched in RelB-abated cells. We first derived a stable HEC-1A cell line in which RelB was knocked down through RNA interference. Two shRNA sequences were used to reduce RelB expression, and with the second shRNA sequence, RelB expression was reduced by approximately 80% at both the mRNA and protein levels (Figure 5a). Consistent with our FCM results, pathway analysis demonstrated that the deregulation of NF-*κ*B, cell cycle and apoptosis signaling was involved in RelB-driven tumorigenesis (Figure5b). Gene Set Enrichment Analysis (GSEA) and hierarchical clustering demonstrated different positive (red) and negative (green) correlation genes associated with these three signals (Figures 5c and d). To assess whether these genes were relevant to the stimulated growth of human EEC cells that endogenously express high levels of RelB, we next examined their mRNA and protein expression patterns in both HEC-1A and RL95-2 cells. Indeed, RelB increased cells exhibited enhanced expression of crucial regulators of the cell cycle transition, proliferation and apoptosis (e.g., c-Myc, cyclin D1, cyclin E1, Bcl-2 and Bcl-xL), whereas negative regulators of cell cycle progression were inhibited (e.g., p21 and p27) (Figures 5e and f).

**Figure 6:**
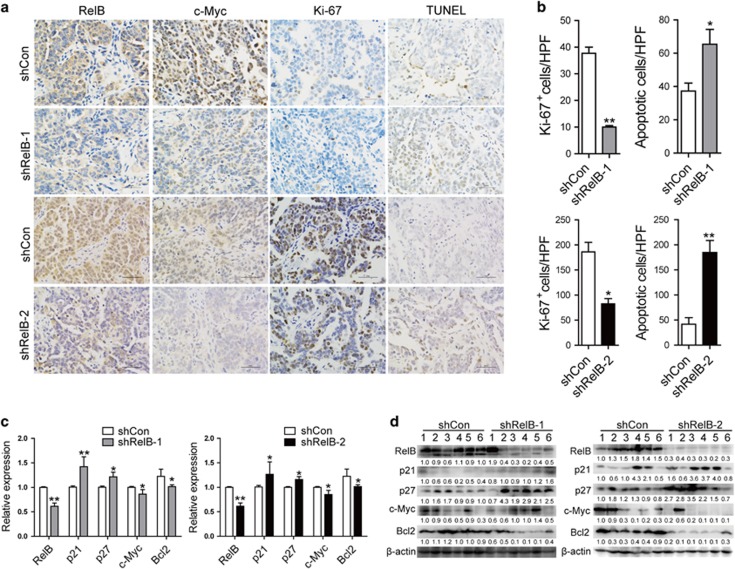
RelB reduction attenuates proliferation and potentiates apoptosis activity *in vivo*. (**a**) IHC staining for RelB, c-Myc and Ki-67 and TUNEL assay results in tumors grafted with HEC-1A cells stably expressing shCon, shRelB-1 or shRelB-2. Scale bars: 50 *μ*m. (**b**) Quantification of Ki-67 IHC staining and TUNEL assay results in tumors with HEC-1A cells stably expressing shCon, shRelB-1 or shRelB-2. The number of Ki-67^+^ or TUNEL-positive cells was averagely estimated in three fields of each section from six mice for each group. (**c** and **d**) Validation of RelB target genes at both mRNA (**c**) and protein (**d**) levels by quantitative real-time PCR and WB in tumors derived from HEC-1A cells stably expressing shCon, shRelB-1 or shRelB-2. Upregulation of p21 and p27 was confirmed; downregulation of c-Myc and Bcl-2 was verified. All data represent the mean±S.D., *P<0.05, **P<0.01

To directly validate the RelB dependency of these core essential genes in tumor cells *in vivo*, subcutaneous grafts derived from HEC-1A cells with reduced RelB expression *versus* empty vector were used to study histological features. Histology revealed that RelB-enhanced tumors exhibited increased Ki-67 (a recognized marker of cell proliferation) and c-Myc staining compared with controls. In contrast, G0/G1 arrest was observed RelB-deficient cells, and terminal deoxynucleotidyl transferase dUTP nick-end labeling (TUNEL) assays exhibited increased apoptosis in response to reduced RelB expression in tumors (Figures 6a and b). Consistent with previously observed molecular events, only 50% of the shRelB-1 tumors demonstrate a downregulation of MYC and in this tumors p21 upregulation is hardly visible. In addition, similar to the *in vitro* model, RelB decreased tumors exhibited p27 upregulation (Figures 6c and d).

Overall, these results confirm that critical genes implicated in cell cycle progression and apoptosis are biologically active and collectively exert pro-proliferative and tumor-initiating activities.

## Discussion

The NF-*κ*B transcription factor complex is involved in various cell processes, such as proliferation, apoptosis and survival.^9, 13, 14, 15^ Recently, numerous studies have documented the role of NF-*κ*B in the initiation, progression and treatment of an extensive range of malignant pathologies. Thus, considerable attention has focused on exploiting the upstream pathways leading to NF-*κ*B activation. For instance, drugs targeting inhibitory kappa B kinases (IKKs), which are required for NF-*κ*B activation, have been developed; however, these drugs are not specific inhibitors of NF-*κ*B transcriptional activity and also have many off-target effects.^16^ Therefore, characterizing the individual role of key NF-*κ*B subunits in cancer may help to overcome these obstacles. In this study, we found that the alternative NF-*κ*B transcription factor RelB is highly upregulated in clinical EC samples, whereas the expression of the canonical NF-*κ*B transcription factor RelA exhibits no significant difference in comparison with normal endometrial specimens. Interestingly, enhanced RelB expression was statistically significant in EEC (type I) but not NEEC (type II), although it is possible that RelB may also show a significant increase in NEEC if a larger sample size is examined. Thus, the role of RelB in NECC should be explored in further studies. Intriguingly, with respect to FIGO staging, significant RelB elevation was observed in the EEC tissues of both stage I and stage II or III, with no important differences noted between the two categorized stages. This finding indicates a crucial role for this transcriptional factor in pre-malignant progression. Taken together, these results suggest that RelB could serve as a possible intervention target for EEC initiation.

Sustaining proliferative signaling and resisting cell death are the hallmarks of cancer.^17^ Indeed, RelB is critical for the G1/S transition and apoptosis evasion of EEC cells, as RelB silencing resulted in the arrest of EEC cells at the G1 phase. Variations in a set of cell cycle-related genes were responsible for these observations; on the one hand, genes encoding cyclin D1 and cyclin E1 were downregulated by RelB inhibition (these proteins accumulate at the G1-S phase boundary and are distinctly conducive to G1/S progression), while on the other hand, cyclin-dependent kinases, which restrain the cell cycle at the G1 phase, were simultaneously upregulated. Coordinated alterations in these genes suggest that key regulators of the cell cycle may function downstream of RelB. Indeed, the expression of c-Myc was positively correlated with RelB expression in EC. C-Myc is an important proto-oncogene that is known to activate the cyclin D and cyclin E complex. Ectopic expression of c-Myc in quiescent cells is sufficient to induce cell cycle entry in the absence of growth factors.^18^ In-depth studies are needed to evaluate the role of c-Myc in RelB-stimulated cell cycle progression, and ectopic expression of c-Myc in RelB-silenced EC cells would help to address this issue.

Anti-apoptosis proceeds partly via the activation of NF-*κ*B.^15^ Paradoxically, RelB knockdown enhanced apoptosis in HEC-1A but not RL95-2 cells, and the RelB depletion impaired Bcl-2 expression was only observed in HEC-1A cells. One possible explanation for this result involves the addition of insulin in the cell culture medium of RL95-2 cells, which is recommended by ATCC, as insulin protects tumor cells from apoptosis. Another explanation for this difference could be the genetic distinction between these two cell lines; RL95-2 cells harbor a complete valine codon 218 deletion in p53, whereas p53 exhibits activity in HEC-1A cells. Cells respond to DNA damage through undergoing apoptotic cell death based on the activation of p53 in various circumstances.^14^ Loss of p53 function may therefore help RL95-2 cells to resist apoptosis induced by RelB reduction. Furthermore, as a transcription factor, RelB directly regulates a broad set of targets, which may differ depending upon the cellular context.

EECs are frequently estrogen receptor *α* (ER*α*)-positive, and decreased expression of ER*α* is noted with disease progression.^6^ Moreover, ER*α* has an inhibitory function on NF-*κ*B activity.^19, 20, 21^ Therefore, it is highly attractive to hypothesize that some correlation may exist between ER*α* and RelB. However, unfortunately, our preliminary experiments revealed no clear relation between these two factors (data not shown), and further investigation into this topic is needed. The alternative NF-*κ*B signaling pathway is activated by multiple upstream factors at different levels, thereby regulating cell proliferation, apoptosis and survival in carcinogenesis.^9^ Nevertheless, our current research failed to explore the stimulated mechanisms upstream of RelB in EEC, which will be further elucidated in our future studies.

In conclusion, the current findings add a new layer of understanding to the complexity of NF-*κ*B subunit activity in tumors. In addition to the classical activation of RelA/NF-*κ*B, more attention should be paid to the noncanonical RelB/NF-*κ*B pathway in specific tumor types. Our results show a previously undescribed function of RelB in the context of EEC and demonstrate that dysfunction of the noncanonical NF-*κ*B pathway is causally associated with this disease setting. Thus, regulation of the cell cycle and apoptosis by RelB in EEC contributes to our knowledge of early tumor development, and drugs specifically targeting RelB may represent a new strategy to combat EC.

## Materials and Methods

### Cell culture and cell lines

The human EC cell lines HEC-1A and RL95-2 were obtained from the American Type Culture Collection (ATCC, Rockville, MD, USA) and maintained under recommended conditions.

### Human samples and immunohistochemistry (IHC)

Two independent cohorts including 333 EC patients were involved in this study. In cohort 1, formalin-fixed, paraffin-embedded (FFPE) tissues from 177 EC patients without clinicopathological factors and 20 noncancerous females were used for first-batch tissue microarray (TMA-1) construction. In cohort 2, FFPE tissues from 156 EC patients with clinicopathological factors and 21 noncancerous controls were used for second-batch tissue microarray (TMA-2) construction. All FFPE tissues obtained between April 2003 and March 2013 were from the Sixth People's Hospital affiliated with Shanghai Jiao Tong University, Shanghai, China; the Changning District Central Hospital of Shanghai, Shanghai, China; and the Huai'an First People's Hospital, Jiangsu, China. All specimens were collected with signed informed consent from all patients, and this study was approved by the local ethics boards and carried out according to the Code of Ethics of the World Medical Association. TMA construction and routine IHC were performed as previously described.^11^ The primary antibodies included RelB (1:100, Santa Cruz Biotechnology, Santa Cruz, CA, USA), RelA (1:100, Santa Cruz Biotechnology), c-Myc (1:100, Cell Signalling Technology, Danvers, MA, USA) and Ki-67 (1:50, Dako, Carpinteria, CA, USA). TUNEL assays were performed using the *In Situ* Cell Death, Fluorescein detection kit (Roche, Basel, Switzerland). The IHC score was evaluated blindly by combining the percentage of staining intensity with positive staining as follows: 0 (negative, no positive cells), 1 (weak, 0–10%), 2 (moderate, 10–60%) and 3 (strong, >60%). The low or high expression groups were denoted as follows: scores of 0 and 1 indicated low expression, and scores of 2 and 3 indicated high expression. The classification of EC was determined according to the criteria proposed by the Bokhman subtype,^2^ and tumor stage was defined based on the FIGO staging system.

### Tumor xenografts

Four-week-old female BALB/c athymic nude mice were purchased from Shanghai Laboratory Animal Center, Chinese Academy of Sciences and Technology (Shanghai, China) and housed under pathogen-free conditions according to the recommendations of Care and Use of Laboratory Animals of the National Institutes of Health. All animal procedures were conducted in compliance with the Guide for the Care and Use of Laboratory Animals and approved by the Institutional Biomedical Research Ethnics Committee of the Shanghai Institutes for Biological Sciences, Chinese Academy of Sciences.

Lentiviral-transduced EEC cells with RelB knockdown *versus* vehicle control (3 × 10^6^ HEC-1A cells or 5 × 10^6^ RL95-2 cells) in 100 *μ*l of medium were hypodermically injected into the left and right scapular regions of the same animal. The tumor diameters were measured every 2 days. Tumor volume was evaluated with the equation V=1/2(*a* × *b* × *b*), where *a* denotes the major tumor axis and *b* the minor tumor axis. The mice were killed at 3–4 weeks post-injection, and dissected tumors were weighed.

### Plasmid construction and cell infection

Three different human RelB-shRNA (short-hairpin RNA) sequences were designed using the RNAi Target Sequence Selector from Clontech (Mountain View, CA, USA) and synthesized by Invitrogen (Carlsbad, CA, USA). shRNA1 and -2 were effective for RelB silencing and were chosen for subsequent experiments. The sequences for shRNA1, -2 and -3 are respectively noted below:

Top strand: 5′-gatccGCAGCAACATGTTCCCCAATTTCAAGAGAATTGGGGAACATGTTGCTGTTTTTTACGCGTg-3′

Bottom strand: 5′-aattcACGCGTAAAAAACAGCAACATGTTCCCCAATTCTCTTGAAATTGGGGAACATGTTGCTGCg-3′

Top strand: 5′-gatccGCGTGCACTAGCTTGTTACATTCAAGAGATGTAACAAGCTAGTGCACGTTTTTTACGCGTg-3′

Bottom strand: 5′-aattcACGCGTAAAAAACGTGCACTAGCTTGTTACATCTCTTGAATGTAACAAGCTAGTGCACGCg-3′

Top strand: 5′-gatccGGAAGATTCAACTGGGCATTTCAAGAGAATGCCCAGTTGAATCTTCCTTTTTTACGCGTg-3′

Bottom strand: 5′-aattcACGCGTAAAAAAGGAAGATTCAACTGGGCATTCTCTTGAAATGCCCAGTTGAATCTTCCg-3′.

Target cells infected with virus-containing supernatant were generated as previously described.^22^ For stable RelB silencing, the cells were screened with 2 *μ*g/ml puromycin. For transient RelB knockdown, the time interval of virus infection was 48–72 h.

### Western blotting (WB) analysis and antibodies

Whole-cell lysates were dissolved in protein lysis buffer, and a protein assay kit (Solarbio Science & Technology Co., Ltd, Beijing, China) was used to determine the concentration of each sample. WB was performed according to a standard protocol. The following antibodies were used: RelB (sc-226, Santa Cruz Biotechnology), p21 (#2947, Cell Signaling), p27 (sc-528, Santa Cruz Biotechnology), cyclin D1 (sc-8396, Santa Cruz Biotechnology), *β*-actin (sc-1616, Santa Cruz Biotechnology), Bcl-2 (sc-7382, Santa Cruz Biotechnology), c-Myc (#5605, Cell Signaling), Bcl-xL (#AB21061, Absci Technology, Shanghai, China), and GAPDH (AB2302, Millipore, Billerica, MA, USA).

### RNA isolation and quantitative real-time PCR

Total RNA from cell lines was isolated with TRIzol reagent (15596-018; Invitrogen) and converted to cDNA using a high-capacity cDNA kit (RR047A; TaKaRa, Shanghai, China). Quantitative PCR for target genes was performed using SYBR Green (RR420A, TaKaRa). Expression was calculated using the 2^−△△Ct^ method as previously described.^22^

### Cell growth, cell counting kit-8 (CCK-8) and colony formation assay

Cell proliferation was analyzed according to growth curves, CCK-8 reagent (CK04; DojinDo, Shanghai, China) and colony formation assays. For growth curves, 1 × 10^4^ cells per well were seeded in a 12-well plate, and cell numbers were assessed each day for 6 days. For the CCK-8 assay, 1 × 10^3^ cells per well were seeded in a 96-well plate, and cell viability was measured as the optical density at 450 nm (OD450) each day for 6 days. For the colony formation assay, 1 × 10^3^ cells or 2 × 10^3^ cells were seeded in 60-mm dishes, and standard culture was performed for 10 days with standard medium renewal. Once completed, visible colonies containing >25 cells were counted after crystal violet staining (C0121; Beyotime, Shanghai, China).

### Flow cytometry (FCM) analysis

Briefly, 1 × 10^5^ HEC-1A or 2 × 10^5^ RL95-2 cells per well were grown in a six-well plate before infection with lentivirus supernatant. After 72 h, the cells were harvested for FCM analysis. For cell cycle analysis, adherent cells were deprived of serum for 12 h for cell cycle synchronization before virus-supernatant infection. The cells were detached, washed and fixed with 70% cold ethanol. After 24 h, cells were treated with 100 mg/l of RNase A (RB0473-100, GeneMarkbio, Taiwan) for 30 min and resuspended in 500 *μ*l of 50 mg/l of propidium iodide (#p4170, Sigma-Aldrich, Biotechnology, St. Louis, MO, USA) for 5 min before FCM analysis. Apoptotic cells were analyzed with Annexin-V-APC (#559925, BD Biosciences, Shanghai, China) and Annexin-V-PE (#556421, BD Biosciences); cells were stained for 30 min before FCM analysis.

### RNA array studies

Human microarrays obtained from Agilent Technologies (Santa Clara, CA, USA) were used in this study. For sample labeling and hybridization, total RNA isolated from HEC-1A cells with transient-silenced RelB *versus* vehicle control was used as the input. The spot intensity values were converted from microarray image information using Scanner Control Software Rev. 7.0 (Agilent Technologies). For normalization and further analysis, background signal subtraction was performed using GeneSpring GX11.0 software (Agilent Technologies, Santa Clara, CA, USA). Hierarchical clustering was used to group genes from RelB knockdown and controls. KEGG pathway analysis and GSEA were performed to identify gene sets and pathways relevant to gene expression data. GSEA (version 2.2.0) (Cambridge, MA, UK) is a powerful analysis tool for integrating gene expression data with gene sets to identify unified biological themes.^23^ Significantly differentially expressed genes were verified by qRT-PCR and WB after identification via Z-score fold-change screening.

### Accession numbers

The Gene Expression Omnibus accession number for the transcriptomics data in this study is GEO: GSE17025.

### Statistical analysis

Statistical analyses were performed using the SPSS version 19 (Chicago, IL, USA) and GraphPad Prism 5.0. (GraphPad Software, Inc., La Jolla, CA, USA) Unless otherwise indicated, the significance of all paired data was determined using the two-tailed Student's *t*-test. The data shown represent the results of experiments performed in triplicate, with error bars representing S.D. or S.E.M. One-way analysis of variance (ANOVA) was performed for quantitative data from GEO DataSets. The *χ* 2 test or Fisher's exact test was performed for enumeration data, and the Mann–Whitney *U-*test or Kruskal–Wallis test was performed for ranked data. Data for GEO DataSets and IHC are presented as scatter dot plots, with a line indicating the mean and 95% confidence interval (CI). A value of *P*<0.05 was considered significant. The following significance values are noted throughout the text: *P<0.05; **P<0.01; **P<0.001.

## Figures and Tables

**Table 1:** The correlation between classified clinicopathological data and ranked RelB expression (*n*=156, TMA-2)

**Variables**	**No. of cases**	**RelB IHC expression**		***P*-value**
		**Low, *n* (%)**	**High, *n* (%)**	
*Age (years)*				0.743
<45	23	2 (1.28)	21 (37.50)	
≥45	133	6 (3.85)	127 (81.41)	
*Histologic subtype*				0.740
EEC	141	8 (51.28)	133 (85.26)	
NEEC	15	0 (0)	15 (9.62)	
*FIGO stage (EEC)*				1.000
S-I	115	7 (4.49)	116 (74.36)	
S-II	12	0 (0)	14 (8.97)	
S-III	14	1 (0.64)	18 (11.54)	
*Pregnancy history*				0.985
No	10	0 (0)	10 (6.41)	
Yes	146	8 (5.13)	138 (88.46)	
*Lymphatic metastasis*				0.952
No	147	7 (4.49)	140 (89.74)	
Yes	9	1 (0.64)	8 (5.13)	
*Vascular invasion*				1.000
No	144	7 (4.49)	137 (87.82)	
Yes	12	1 (0.64)	11 (7.05)	
*Menopause*				0.918
No	71	3 (1.92)	68 (43.59)	
Yes	85	5 (3.21)	80 (51.28)	

## Abbreviations

EEC: endometrioid adenocarcinoma
NEEC: non-endometrioid adenocarcinoma
RelB: avian reticuloendotheliosis viral (v-rel) oncogene related B
Bcl-3: B-cell CLL/lymphoma 3
p27: cyclin-dependent kinase inhibitor 1B
p21: cyclin-dependent kinase inhibitor 1A
c-Myc: v-myc avian myelocytomatosis viral oncogene homolog
TUNEL: terminal deoxynucleotidyl transferase-mediated deoxyuridine triphosphate nick-end labeling
IHC: immunohistochemistry
FFPE: formalin-fixed, paraffin-embedded
TMA-1: first-batch tissue microarray
TMA-2: second-batch tissue microarray
FIGO: the International Federation of Gynecology and Obstetrics
GSEA: Gene Set Enrichment Analysis
shRNA: short-hairpin RNA
ER*α*: estrogen receptor *α*
