# Supplementary material for: RelB/NF-κB links cell cycle transition and apoptosis to endometrioid adenocarcinoma tumorigenesis
Source: Cell Death Dis. 2016 Oct 6;7(10):e2402–. doi: 10.1038/cddis.2016.309 (PMC5133976; doi:10.1038/cddis.2016.309)
Supplement: Supplementary Figure Legend [file cddis2016309x1.doc]

**Supplementary Figure Legends**

**Figure S1. Other NF-κB signaling pathways are not regulated in the RelB knock-down EEC cells.**

**a**-**c** The total (a), cytoplasmic (b) and nuclear (c) protein levels of RelA, NF-κB1, NF-κB2 or c-Rel in the RelB knock-down HEC-1A and RL95-2 cells.
