# Supplementary figures and images for: RelB/NF-κB links cell cycle transition and apoptosis to endometrioid adenocarcinoma tumorigenesis
Source: Cell Death Dis. 2016 Oct 6;7(10):e2402–. doi: 10.1038/cddis.2016.309 (PMC5133976; doi:10.1038/cddis.2016.309)

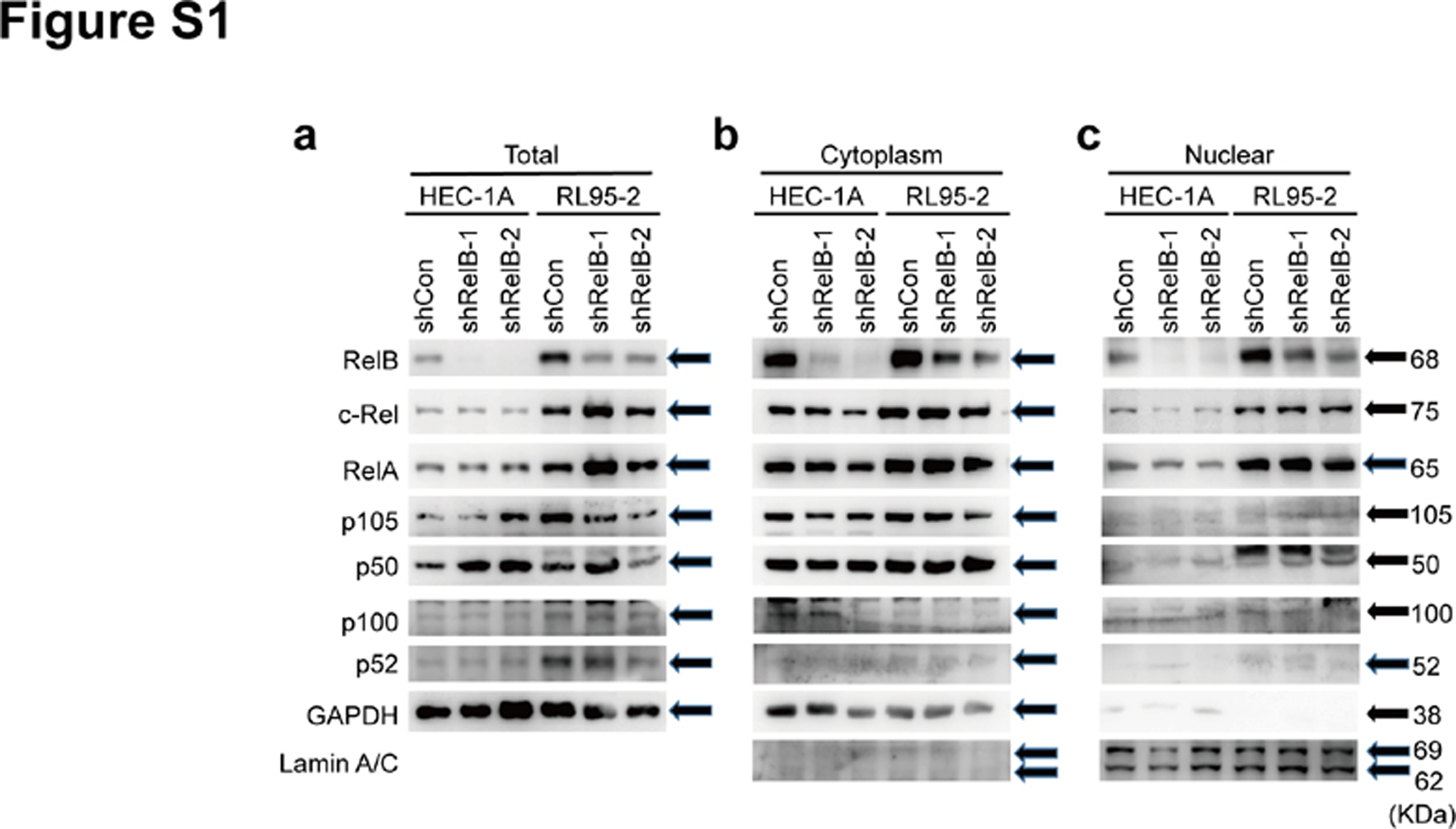

Supplement: Supplementary Figure 1 [file cddis2016309x2.tif]
